# Molecular signatures of silencing suppression degeneracy from a complex RNA virus

**DOI:** 10.1371/journal.pcbi.1009166

**Published:** 2021-06-28

**Authors:** Silvia Ambrós, Neus Gómez-Muñoz, Silvia Giménez-Santamarina, Javier Sánchez-Vicente, Josep Navarro-López, Fernando Martínez, José-Antonio Daròs, Guillermo Rodrigo

**Affiliations:** 1 Centro de Protección Vegetal y Biotecnología, Instituto Valenciano de Investigaciones Agrarias (IVIA), Moncada, Spain; 2 Instituto de Biología Molecular y Celular de Plantas (IBMCP), CSIC–Universitat Politècnica de València, València, Spain; 3 Instituto de Biología Integrativa de Sistemas (I2SysBio), CSIC–Universitat de València, Paterna, Spain; University of Ottawa, CANADA

## Abstract

As genomic architectures become more complex, they begin to accumulate degenerate and redundant elements. However, analyses of the molecular mechanisms underlying these genetic architecture features remain scarce, especially in compact but sufficiently complex genomes. In the present study, we followed a proteomic approach together with a computational network analysis to reveal molecular signatures of protein function degeneracy from a plant virus (as virus-host protein-protein interactions). We employed affinity purification coupled to mass spectrometry to detect several host factors interacting with two proteins of *Citrus tristeza virus* (p20 and p25) that are known to function as RNA silencing suppressors, using an experimental system of transient expression in a model plant. The study was expanded by considering two different isolates of the virus, and some key interactions were confirmed by bimolecular fluorescence complementation assays. We found that p20 and p25 target a common set of plant proteins including chloroplastic proteins and translation factors. Moreover, we noted that even specific targets of each viral protein overlap in function. Notably, we identified argonaute proteins (key players in RNA silencing) as reliable targets of p20. Furthermore, we found that these viral proteins preferentially do not target hubs in the host protein interactome, but elements that can transfer information by bridging different parts of the interactome. Overall, our results demonstrate that two distinct proteins encoded in the same viral genome that overlap in function also overlap in their interactions with the cell proteome, thereby highlighting an overlooked connection from a degenerate viral system.

## Author summary

Determining the mode of action of plant viruses (*i*.*e*., the interactions that they establish with their host plants) is the cornerstone of modern molecular virology. In this work, a proteomic study was conducted to determine protein complexes that associate with two proteins of *Citrus tristeza virus* (citrus tristeza virus, CTV): p20 and p25. CTV is a plant virus of agronomic importance that also serves as a suitable model of viral complexity. p20 is a silencing suppressor that acts at both local and global levels, while p25 is the coat protein of the virus and also functions as a systemic silencing suppressor. Computational analyses revealed that CTV evolved different silencing suppressors that can establish convergent virus-host interactions, which is a mark of protein function degeneracy.

## Introduction

**Fig 1:**
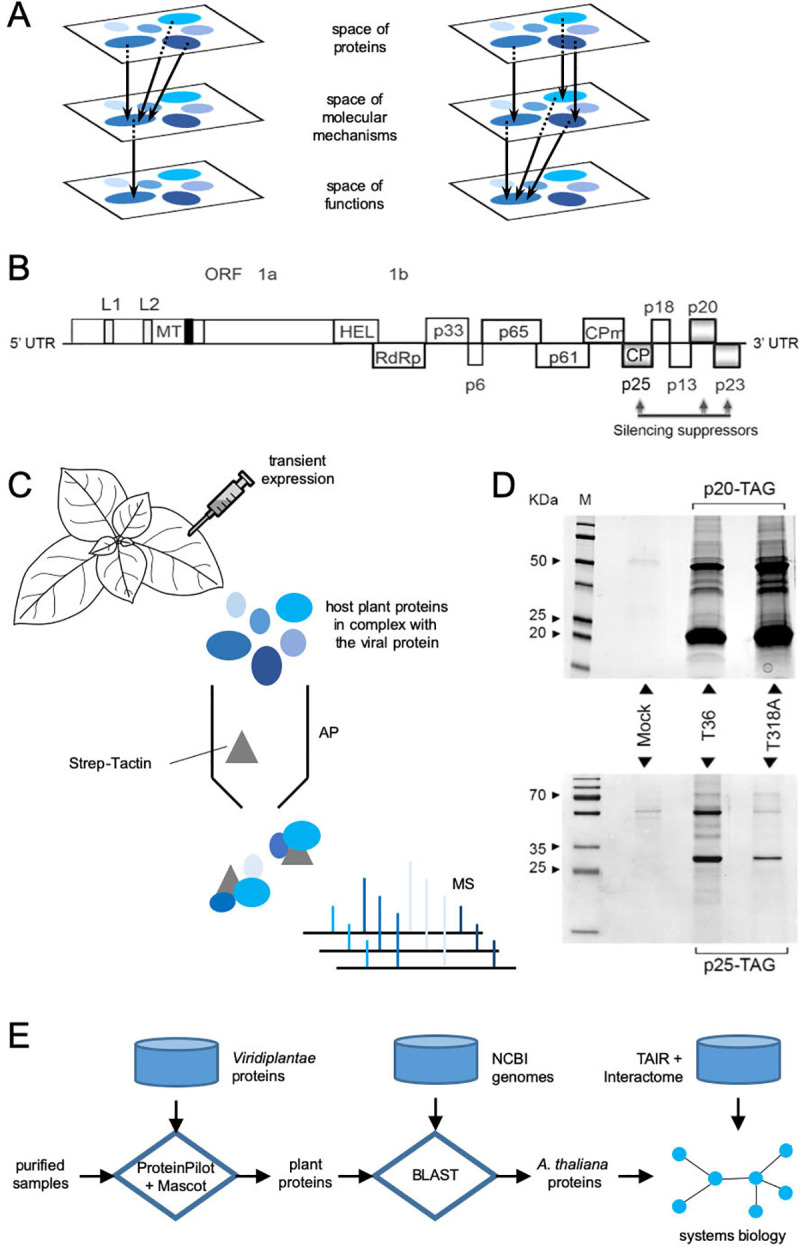
A) Schematic representation of protein function degeneracy. Left: overlap in function accomplished through an overlap in molecular interactions. Right: overlap in function accomplished through different molecular interactions. B) Schematic representation of the genome architecture of CTV. This shows its different proteins, including the two objects of this study (p20 and p25), which are two known suppressors of RNA silencing. C) Schematic representation of the adopted experimental framework: affinity purification (AP) followed by mass spectrometry (MS) from transient expression in plants of the viral protein of interest tagged with the Twin-Strep-tag. D) Electrophoretic assay (western blot) to confirm the transient expression of the tagged p20 and p25 in plants. The sequences from two viral isolates (T36 and T318A) were considered. E) Schematics of the computational pipeline used to analyze the AP-MS data.

Modern natural genomic architectures are the result of millions of years of evolution that have subjected them to a tinkering process with no rational *a priori* direction [1,2]. It would then not be surprising that such architectures could accommodate genetic architecture features with no clear meaning (function). One such feature is genetic degeneracy, which is manifested when two or more different structural elements (*e*.*g*., proteins) overlap in function in certain contexts (**Fig 1A**) [3,4]. Beyond the evolutionary paradigm of providing mutational robustness and/or increased evolvability [5,6], recent studies exploiting information theory frameworks have suggested the idea that genetic degeneracy (or also genetic redundancy, when the structural elements are identical) is instrumental for organisms to operate with greater precision [7–10]. The resulting networks provide alternative information flow channels to buffer fortuitous errors in protein expression due to intrinsic molecular noise [11] or errors in protein activity due to large-scale molecular interaction rearrangements [12]. Even though, degeneracy is a genetic architecture feature that has been long assumed to go in parallel with complexity [3], so the occurrence of degeneracy remains elusive in minimal genomes, such as those of viruses. These genomes are highly constrained in size and evolve rapidly as a consequence of high mutation rates [13]. A plausible scenario would be that degeneracy contributed to achieve better adaption to new hosts by the virus. This is because in such systems there is higher freedom to functionally diverge over time, and then the probability of finding genetic solutions to efficiently perform in different environments increases, as previous theoretical work pointed out [6]. An alternative, yet complementary scenario would be that degeneracy provided *per se* a selective advantage for the virus in the same environment as a result of the establishment of several congruent information flows, such as to surmount the host defense machinery more efficiently [14]. However, without a precise characterization of the mechanistic mode of action of the different structural elements at play (*e*.*g*., through virus-host proteomics), it is difficult to derive any conclusion.

In this work, we considered *Citrus tristeza virus* (citrus tristeza virus, CTV) as a model system to depict molecular signatures of protein function degeneracy from a virus. Notably, CTV is a plant virus of agronomic importance [15]. Belonging to the family *Closteroviridae*, CTV is one of the largest monopartite RNA viruses found in nature (the largest in plants), with a genome of approximately 20 kilobases that contains 12 open reading frames (**Fig 1B**) [15]. More in detail, CTV encodes three different RNA silencing suppressors: p20, p23, and p25 [16]. In terms of function, p20 is similar to the suppressor 2b of *Cucumber mosaic virus* because it blocks intercellular (systemic) and intracellular RNA silencing and is also responsible for the formation of inclusion bodies during infection [16,17]. p23 is similar to the suppressor HC-Pro of potyviruses and inhibits RNA silencing at the single cell level. p23 also modulates viral RNA synthesis and acts as a determinant of pathogenesis [16,18]. p25 is the coat protein of CTV, which also functions as a weak intercellular silencing suppressor [16,19]. Consequently, we followed a proteomic approach to study the mode of action of p20 and p25 (*i*.*e*., to determine which proteins they interact with in the host plant). Notably, this problem remains largely unaddressed in the case of CTV. We chose these two proteins because they can suppress RNA silencing from a long distance and also for simpler comparative analysis. Broadly, we aimed to determine whether an overlap in function within a natural degenerate system is accomplished through an overlap in molecular interactions or through entirely different modes of action (**Fig 1A**). To expand the analysis, we considered two CTV isolates that differ in their induced symptomatology in citrus and infectivity in *Nicotiana benthamiana* [20]. In particular, the quick decline isolate T36 from Florida [21] and the stem pitting isolate T318A from Spain [22] were considered, both completely sequenced. As rapidly evolving agents, viruses display highly variable genomic sequences.

## Results

### Virus-host protein interactome identification

We began with the transient expression of the four viral proteins of interest (the two p20s and two p25s from the CTV isolates T36 and T318A) in *N*. *benthamiana* plants using agroinfiltration (**Fig 1C**). Although this is a non-natural host of CTV, it is suitable for controlled experimental developments and also susceptible to be systemically infected by this virus (isolate T36) showing characteristic disease symptoms [20]. Hence, we first amplified the gene sequences from two infectious clones corresponding to the two isolates [20], and then they were tagged at the C-terminal with the synthetic peptide Strep. **S1 Table** displays an alignment between the proteins of both isolates (EMBL-EBI web services), showing that the two p20s differ in five amino acids, while the two p25s differ in nine amino acids. The tagged viral proteins and the host plant proteins that formed complexes with them were purified by affinity chromatography, and the resulting samples were analyzed by mass spectrometry to obtain lists of interacting proteins (**Fig 1C**). The expression of the tagged viral proteins *in planta* was confirmed by western blot analysis (**Fig 1D**). Moreover, we provide experimental evidence of the tagged viral proteins exhibiting silencing suppression activity in *N*. *benthamiana* plants similarly to their native counterparts (**S1 Fig**).

Affinity purification coupled to mass spectrometry (AP-MS) is a powerful proteomic technique that has been successfully exploited to decipher protein-protein interactions between plant or animal viruses and their respective hosts [23,24], as well as protein-protein interactions in many other contexts, especially when protein lists are appropriately filtered out from background contaminants [25]. We achieved this by performing control experiments with no viral protein, which allowed us to remove proteins that systematically appear in the purification and thus do not interact specifically with p20 and p25, and also by using a MS-derived quantitative index of protein amount in the samples [26]. Subsequently, we mapped the lists of retrieved proteins from the subkingdom *Viridiplantae* (green plants) to proteins of the model plant *Arabidopsis thaliana*. It is presumed that ortholog proteins between plants behave similarly. Although CTV does not infect *A*. *thaliana*, this mapping allowed us to perform successive functional and network analyses from a systems biology perspective (**Fig 1E**) while taking advantage of all available annotations [27]. To this end, we developed a computational procedure to automatically map proteins between two arbitrary organisms (*i*.*e*., by reading the genomes of the source and target organisms and performing on the fly different sequence alignments; **S2 Fig**). Here, the source organisms given by the MS search engine were from *Viridiplantae*, and the target organism was always *A*. *thaliana*. **S1 Data** contains the different protein lists (raw, mapped, and filtered). Notably, since AP-MS detects protein complexes, we could expect that some of the identified host factors interacted with the viral proteins through third parties.

### Analysis of degeneracy in the identified interactome

**Fig 2:**
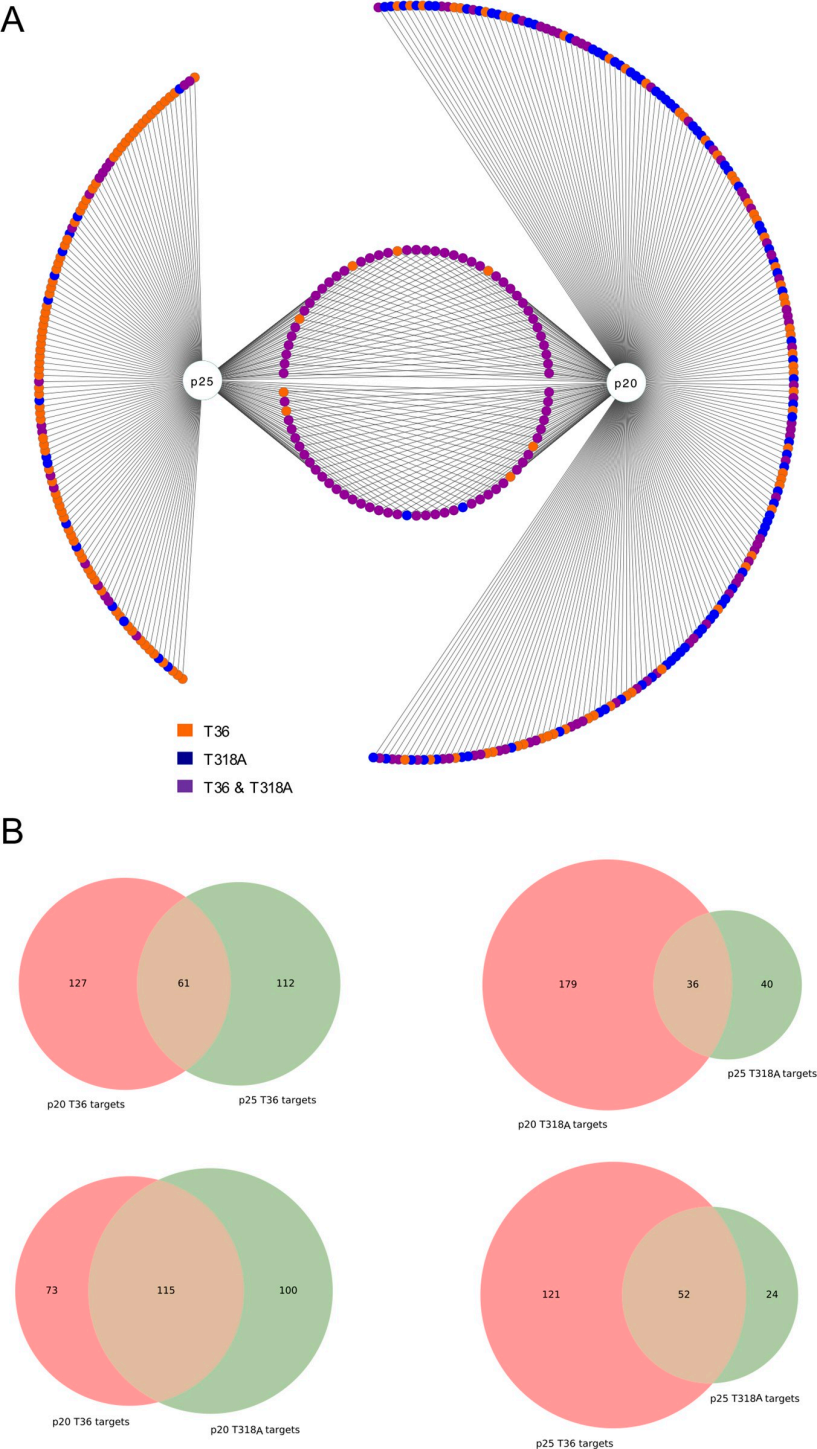
A) Illustration of the protein targets identified by AP-MS of the CTV proteins p20 and p25. Note that target proteins are mapped to *A*. *thaliana* genes. Colors indicate the CTV isolate (T36 or T318A) from which the target was identified. For the purpose of representation, common interactors of p20 and p25 are colored in purple if they were identified from both isolates of at least one viral protein. B) Venn diagrams between the different protein targets identified by AP-MS (two-set comparisons).

We found that p20 putatively interacts with 288 plant proteins and p25 interacts with 197 (**Fig 2**; see also **S2 Data**). This corresponds to a merged picture of the identified interactions from the AP-MS analysis having used the variants from two isolates. Among them, we identified 86 plant proteins that interact with both viral proteins. This is a substantial set of common interactors that illustrates, given that p20 and p25 share a function, a partial overlap in terms of their interaction with the host, which presumably results in an overlap of the molecular mechanisms followed by both proteins to achieve that shared function. This contributes to supporting the notion of protein function degeneracy (silencing suppression degeneracy, in this case) in CTV (a scenario in between the two depicted in **Fig 1A**). Interestingly, most of the shared interactors are associated with both isolates (Fisher’s exact test, *P* < 0.0001), which highlights that such overlap diverged little from one isolate to another. Among this common set, we found 41 chloroplastic proteins (*e*.*g*., a S-adenosylmethionine carrier [SAMC1; locus AT4G39460] and a photosystem reaction protein [PSBA; locus ATCG00020]) and four translation factors (*e*.*g*., an elongation factor of the Tu family [EF1α; locus AT5G60390]). Interestingly, some viral proteins have been shown to interact with chloroplastic proteins to modulate the plant immune system during infection [28,29]. We also found a glyceraldehyde 3 phosphate dehydrogenase (GAPC1; locus AT3G04120) as a common interactor, which has been reported to interact with p23 [30].

To give further support to the reported overlap between p20 and p25, we considered the putative interactors of *Tobacco etch virus* VPg, which were also obtained by AP-MS in previous work [23]. VPg is a potyviral protein involved in replication that is known to interfere with the RNA silencing machinery, thereby functioning as a secondary silencing suppressor [31]. We found that VPg shares 50 interactors with p20 and 56 interactors with p25 (considering both isolates). Furthermore, we found that 32 out of the 86 common interactors of p20 and p25 also associate with VPg (including heat-shock, chloroplastic, and translation-related proteins), while the 63% of the overlap shown in **Fig 2** appears specific to the two CTV proteins (**S3 Fig**).

In addition, we observed that most of the specific targets of p25 (111 plant proteins, mainly related to different metabolic processes and cell structure, such as actins or tubulins) were detected only upon expression of the T36 variant (Fisher’s exact test, *P* < 0.0001). In particular, we found RAN GTPase 3 (RAN3; locus AT5G55190), a plant protein involved in import movement into the nucleus, to interact with p25 (from isolate T36), which agrees with recent results showing that this viral protein localizes in the nucleus [32]. In contrast, most of the specific targets of p20 (202 plant proteins, mainly related to transport and localization, but also metabolism) were detected only upon expression of the T318A variant (Fisher’s exact test, *P* < 0.0001). However, we observed two significant interactions related to its main function: p20 (from both isolates) targets argonaute 1 (AGO1; locus AT1G48410) and argonaute 2 (AGO2; locus AT1G31280), which are key components of the RNA silencing machinery that bind small interfering RNAs (siRNAs) to form the RNA-induced silencing complex (RISC) and degrade the genomic RNA of the virus [33]. Conversely, if we control for isolate, 300 plant proteins interact with the viral proteins of T36, while 255 interact with those of T318A. This result suggests that if a given protein loses interactors during viral sequence divergence (from T36 to T318A, p25 lost 97 interactors), the other gains a counterpart (from T36 to T318A, p20 gained 27 interactors). Collectively, these results suggest that p20 and p25 inhibit systemic RNA silencing by concurrent specific and common mechanisms at the molecular level.

### Functional analysis of host targeted proteins

**Fig 3:**
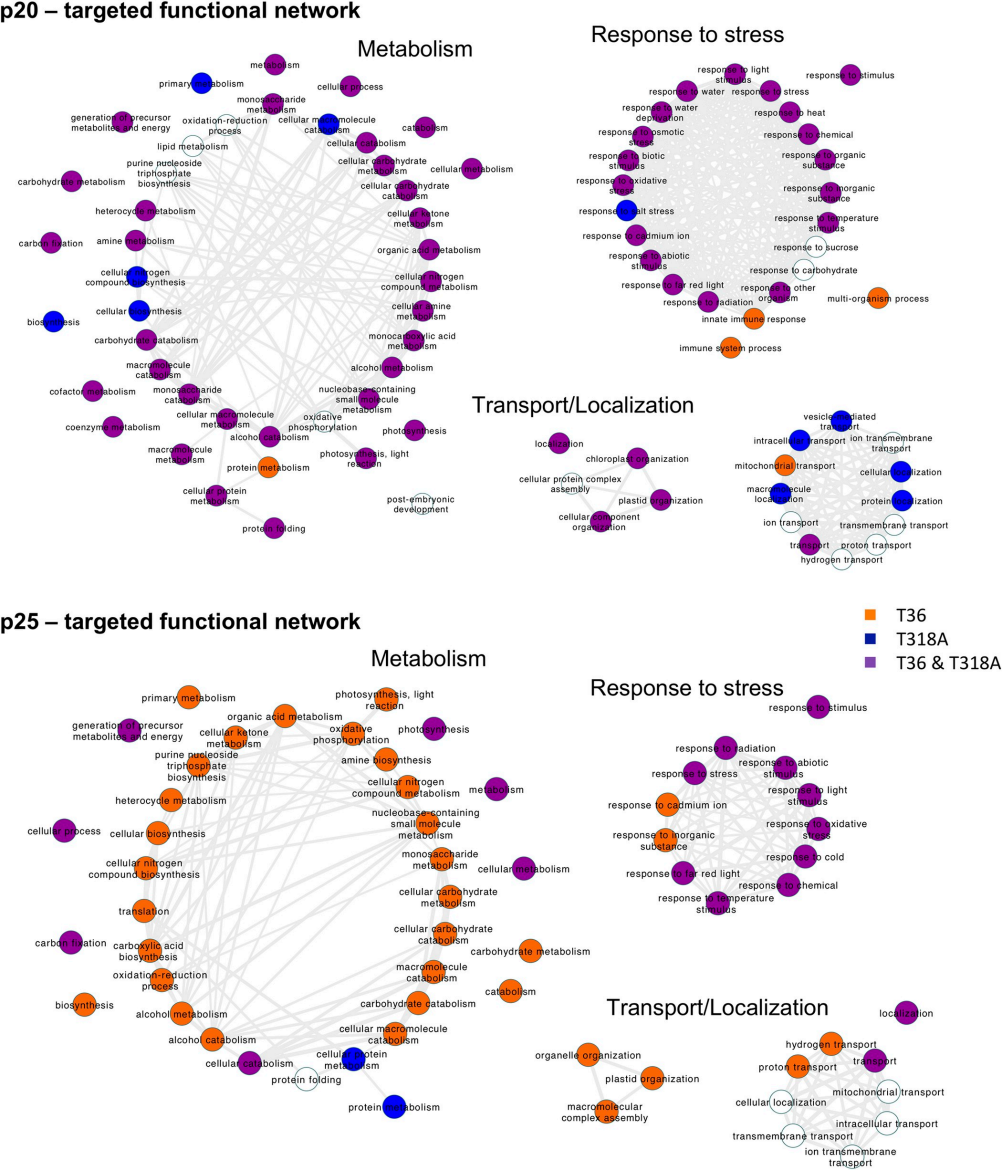
Functional analysis of the protein targets identified by AP-MS. Top: GO network for the CTV protein p20. Bottom: GO network for p25. Three main independent functional modules are shown (functions sharing elements are connected). Colors indicate the CTV isolate (T36 or T318A) from which the target was identified. White terms only appear in the functional analysis when all interactors, irrespective of the isolate, are merged.

To further analyze the mode of action of the two viral proteins under study, we performed a computational analysis of the function of their targets. To achieve this, we applied gene ontology (GO) over the identified host targets to assess the enrichment in different biological processes [34]. This was performed separately for the interactors of p20 and those of p25, while controlling for the contribution of each isolate (**Fig 3**; see also **S3 Data**). Broadly, we observed three different modules that were congruent with the two viral proteins. The first corresponds to metabolism, while the second corresponds to response to stress, and the third corresponds to transport and localization. This suggests that both viral proteins touch host proteins that have very similar functions in the cell despite not necessarily being the same molecule. For example, p20 (from both isolates) targets argonaute 4 (AGO4; locus AT2G27040), which is a key component of the RNA silencing machinery for the plant DNA methylation [33,35]. Yet, p25 (only from isolate T36) targets an S-adenosylmethionine synthetase (SAM2; locus AT4G01850) and an S-adenosyl-L-homocysteine hydrolase (SAH1; locus AT4G13940), which are also required for methylation [36,37]. Interestingly, SAH1 was also reported as a target of HC-Pro [38]. Additionally, both viral proteins target four different heat-shock proteins (*e*.*g*., the chloroplast heat-shock protein 70–2; locus AT5G49910). However, the network associated with p20 contains more GO terms (also because this viral protein has more interactors), which indicates that p20 introduces a greater perturbation on plant physiology. In particular, 24 terms of response to stress were recognized as perturbed upon p20 expression, while only 12 were recognized upon p25 expression. Furthermore, we observed that nearly all functional terms participated by the proteins targeted by p20, especially those related to metabolism and response to stress, were associated with both isolates. This suggests that p20 slightly diverged in function from one isolate to another. Nevertheless, this is not the case for the functional network of the proteins targeted by p25. While nearly all metabolic terms are only associated with isolate T36 in this case, the response to stress seems to be shared. Consequently, it is possible that p25 greatly diverged in function (perhaps by losing some dispensable subfunctions) from T36 to T318A.

### Global contextualization of host targeted proteins

**Fig 4:**
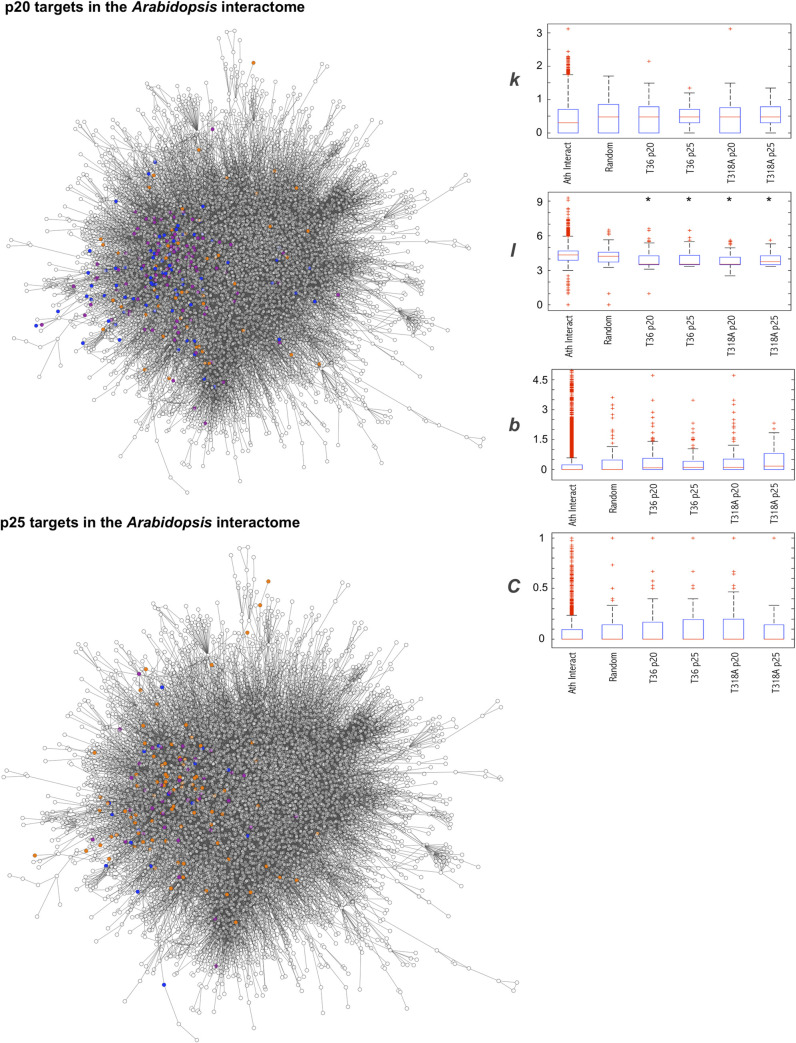
Contextualization of the protein targets identified by AP-MS in the experimental *A*. *thaliana* interactome. Illustration of the global interactome together with a boxplot of the global topological properties (degree [*k*], average shortest path length [*l*], betweenness centrality [*b*], and clustering coefficient [*C*]) of the CTV targets. Top: targets of CTV protein p20. Bottom: targets of p25. Note that these properties were calculated according to the global interactome and not to a particular subnetwork. *Statistical significance (Mann-Whitney’s *U*-tests, *P* < 0.001). The distributions of a representative random sample and the whole interactome are shown as controls. Colors indicate the CTV isolate (T36 or T318A) from which the target was identified.

Next, we contextualized the identified host targets on the protein interactome of *A*. *thaliana* (**Fig 4**), which was essentially developed by yeast two-hybrid experiments [39,40]. This interactome covers approximately 8,000 plant proteins, has approximately 22,000 non-redundant interactions between them, and is a useful tool for studying the mode of action of viruses (and also other stresses) in plants [41]. Previous results on virus-host interactions (mainly in animals) have suggested that host targets are preferentially highly connected elements (hubs) within the interactome [42,43], which should translate into a major impact on host physiology. However, recent studies have pointed out that some pathogen proteins can instead preferentially attack elements that bridge different parts of the network with sufficient connectivity and that are closer to its core (*i*.*e*., not necessarily hubs) [44]. Here, we did not find significant differences with respect to the null distributions (for random lists of proteins or the whole interactome) in terms of degree of connectivity, betweenness, and clustering for both p20 and p25 (Mann-Whitney’s *U*-tests, *P* > 0.05; boxplots in **Fig 4**). In contrast, differences were significant in terms of average shortest path length (Mann-Whitney’s *U*-tests, *P* < 0.001) for both p20 and p25. In particular, the host targets connect on average in 3.85–3.95 steps (depending on the CTV protein and isolate considered) with any other protein of the interactome, while randomly chosen proteins connect in 4.30 steps. This suggests that the local perturbations induced by these viral proteins (*e*.*g*., inhibition upon interaction of the function of a host factor involved in response to stress, such as AGO1, with an average shortest path length of 3.50) might easily have a more global impact. Although AGO1 is not a hub in the interactome, it does interact with highly connected proteins (neighborhood connectivity of 352). This adds to the fact that protein function degeneracy is presumed to enhance information transfer throughout the entire pathosystem [7–10].

### Degeneracy in a molecular interaction cluster

**Fig 5:**
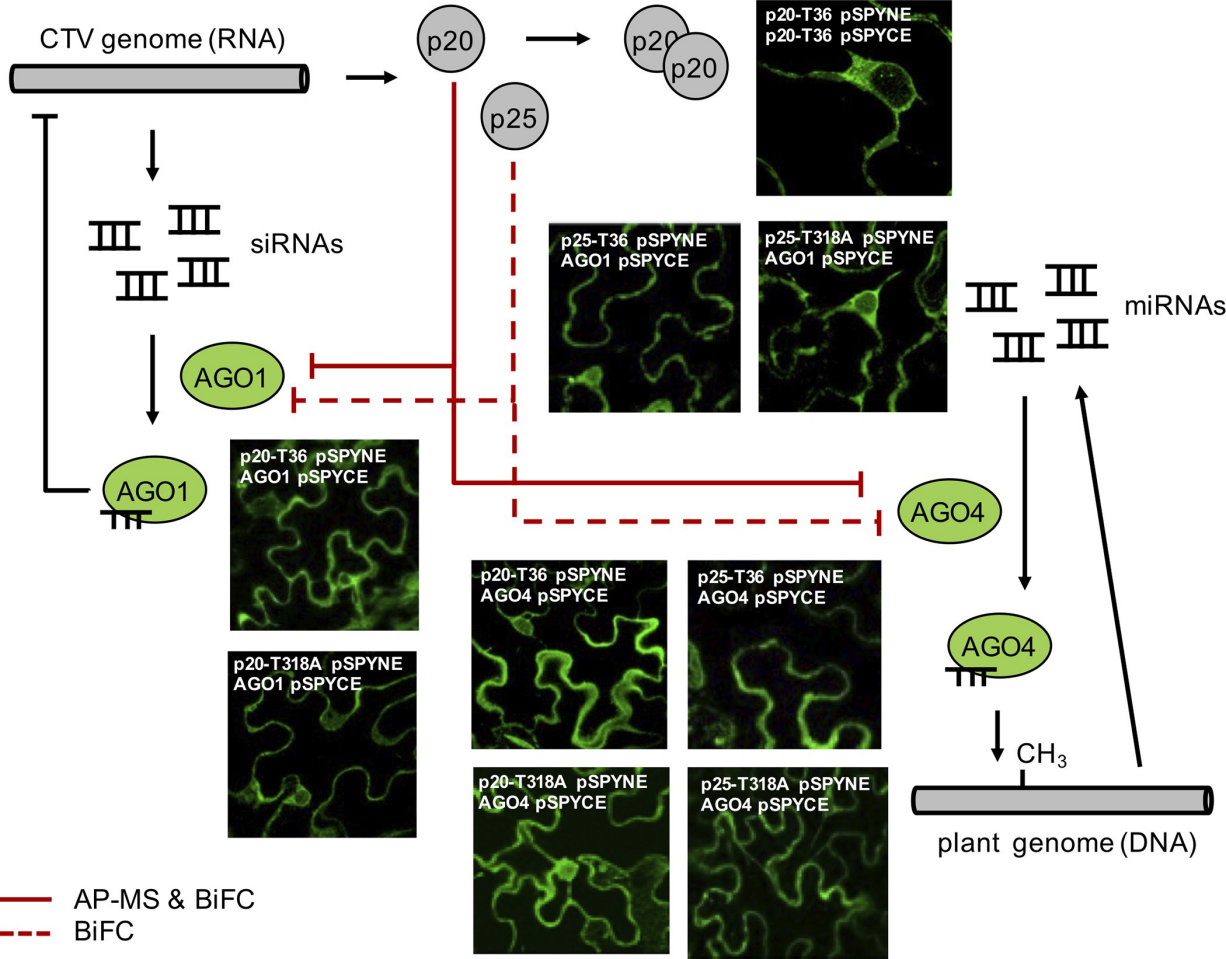
Schematic representation of the regulatory circuit established between p20 and p25 (from the virus) and AGO1 and AGO4 (from the plant) in which patterns of degeneracy are manifested, together with images of BiFC assays *in planta* by using a split YFP system to prove the virus-host protein-protein interactions.

According to our AP-MS results, AGO1 and AGO4 are specific interactors of p20 (from both isolates). Intriguingly, the family of argonaute proteins, with up to 10 different elements, constitutes a degenerate defense system in plants, although some of them have reached a high level of specialization [33,45]. In infections with RNA viruses, AGO1 associates with siRNAs to degrade the viral genome, while AGO4 associates with endogenous small RNAs to modulate the expression of host genes through methylation and then promote the defense response [33]. Thus, these two Argonaute proteins present distinct roles in antiviral defense. Due to their relevance, we decided to further characterize these interactions via bimolecular fluorescence complementation (BiFC) assays [46] in *N*. *benthamiana* plants using a split yellow fluorescent protein (YFP) [47] to gain mechanistic insights. BiFC assays confirmed the p20-AGO1 and p20-AGO4 interactions; however, surprisingly they revealed that these two argonaute proteins are also interactors of p25 (**Fig 5**). These latter interactions escaped our AP-MS approach, which may indicate that these argonaute proteins interact more weakly with p25 than with p20. As a control experiment, we used BiFC assays to assess the interaction of CTV with thioredoxin H-type 9 (TH9; locus AT3G08710), an element involved in oxidative stress and cell-cell movement [48]. According to our AP-MS results, TH9 is a specific interactor of p25 that was only detected when expressing the T318A variant. Notably, BiFC assays successfully confirmed this interaction profile (**S4A Fig**) and also verified that our results are not a consequence of spurious interactions (*e*.*g*., due to self-assembly of the two YFP moieties; **S4B Fig**). These results illustrate how viruses (through their respective silencing suppressors) have multiple means to disrupt the different RISC-mediated channels in the host that evolved to deploy a sophisticated antiviral response.

## Discussion

To achieve at least partial systemic RNA silencing suppression, it would be expected that a viral protein must touch several elements of the host since this mechanism involves multiple interlinked routes for the biogenesis, amplification, and transport of siRNAs [33,49]. Our results reveal that p20 and p25 putatively interact with multiple plant proteins. Since some of these are common, it suggests a partial overlap in their mechanistic mode of action. Arguably, some of the specific interactors of each viral protein contribute to an exclusive task (*e*.*g*., virion formation in the case of p25) [16]. Remarkably, we found strong evidence of argonaute proteins AGO1 and AGO4 being relevant targets of p20 since they appeared in AP-MS and BiFC assays. This agrees with recent results suggesting that CTV suppressors act once the siRNAs are generated [50]. Notably, the functionally-related suppressor 2b of *Cucumber mosaic virus* has been shown to target AGO1 and disrupt its cleavage ability [51]. Furthermore, our results reveal that certain specific interactors of p20 display functions in the plant that are very similar to the functions displayed by some specific interactors of p25, which may indicate a greater overlap in terms of silencing suppression. A global network analysis also suggests that the mode of action of these viral proteins is through bridging points in all cases. Together, this leads us to suggest that CTV evolved different silencing suppressors to better surmount the plant antiviral response from convergent virus-host interactions (*i*.*e*., interactions that are established between different viral proteins and the same host protein).

Besides, it is important to note the difference between degeneracy and multifunctionality [52]. Viral proteins are multifunctional elements that facilitate the efficient completion of the infectious cycle (*e*.*g*., HC-Pro is involved in genome replication and silencing suppression in potyviruses, while p25 is the coat protein and a silencing suppressor in CTV) [53]. In principle, multifunctionality does not entail degeneracy, unless a common function is performed by the different proteins (*i*.*e*., one protein can be somewhat dispensable). In this regard, the aforementioned situations are quite different since HC-Pro cannot replicate the genome by itself (no degeneracy, but cooperation occurs with the viral RNA-dependent RNA polymerase) [54], while p25 can suppress RNA silencing when expressed individually (degeneracy, together with p20 and p23) [16,19]. Moreover, we must note that p20 and p25 are not functionally redundant, since a CTV mutant with deleted p25 exhibits replication defects in protoplasts, while a mutant with deleted p20 only shows defects in systemic infection.

Interestingly, the occurrence of degeneracy in viral genomes, as it is the case of p20, p23, and p25 in CTV, might contribute to enlarging the host range and codifying host specificity. In recent work with *Dengue virus*, a duplication of an RNA motif in the 3’ untranslated region was shown to provide host specificity [55]. In CTV, the non-conserved genes p13, p18, and p33 have also been highlighted as required for infecting different hosts [56]. Alternatively, without invoking an increased ability of the virus to adapt to new hosts, degeneracy might facilitate increased performance in the same environment since multiple proteins with overlapping functions can lead to better information processing and to reach robustness. From a population genetics perspective, this might explain why different genes that can be dispensable in some hosts are fixed in the viral genome [11]. Future work should perform proteomic studies in multiple hosts to assess how the virus-host network changes with the aim of identifying general and specific interactions.

Moreover, we might speculate that the divergence between T36 and T318A is at least partially responsible for the differential infectivity observed between both isolates in *N*. *benthamiana* [20]. Only isolate T36 can systemically infect this plant, and we found numerous additional interactors for p25 from T36. Infectivity is generally greater when the RNA and protein degradation pathways of the host are affected [57]. According to our AP-MS results, the hydrolase SAH1 for methylation-dependent silencing and the proteasome subunit PBG1 (locus AT1G56450) are two global host proteins targeted by p25 from T36 but not by p25 from T318A. Hence, a higher accumulation of the virus in the cell would be expected upon targeting these degradation pathways. Another crucial step for successful infection concerns the intercellular movement. In this regard, we observed that p25 only from T36 targets various actin depolymerizing factors (*e*.*g*., ADF2, locus AT3G46000), which might subvert the actin cytoskeleton to promote the viral spread [58]. Isolate T318A may then face more difficulties to accumulate and move through the plant by not targeting these host proteins. Moreover, we found that a pathogenesis-related protein (PR4; locus AT3G04720) interacts uniquely with p25 from T318A. Pathogenesis-related proteins are central, salicylic acid-mediated plant defense factors that are over-expressed in response to viral infections through the control of non-expresser of PR genes 1 (NPR1; locus AT1G64280). Arguably, PR4 targets the viral protein to prevent the systemic infection of the virus [59]. The interaction between TH9 and p25 from T318A is also suggestive because the oligomer-to-monomer reaction that changes the transcriptional activity of NPR1 is catalyzed by thioredoxins [60]. In this regard, isolate T318A may not escape the surveillance of the NPR1 pathway (systemic resistance), then exhibiting limited infectivity.

In addition, the symptoms induced in citrus by these two isolates are different. A quick decline in tree health upon infection with isolate T36 might be aligned with the fact that p25 from such isolate targets a substantially greater number of metabolic pathways when compared to p25 from T318A. In turn, yellow leaf disorder upon infection with both isolates would be the result of targeting a series of chloroplastic proteins.

The present study presents nonetheless certain limitations. One limitation is that *A*. *thaliana* is not a natural host of CTV. We mapped the AP-MS hits to *A*. *thaliana* orthologs to perform subsequent computational analyses by exploiting functional annotations and protein networks for this model plant [27,39]. Thus, our results should be interpreted with care since the precise functioning and regulation of the real citrus proteins targeted by CTV might be different. Another limitation is that an experimental approach of transient expression does not reflect the actual expression level of the infection context, which can even change with space and time, despite it allowing the identification of a full list of putative interactors. Ultimately, the present work contributes to further highlighting the ubiquity of genetic degeneracy [3], even in simple biological entities such as viruses, and provides evidence of a sophisticated function being performed by different proteins that result from of specific and common molecular mechanisms. This pattern is expected to be pervasive in degenerate viral systems that require the inactivation and/or hijacking of multiple host factors.

## Methods

### Plasmid construction

To perform the AP-MS study, CTV genes p20 and p25 were amplified by polymerase chain reaction (PCR) from the full-length T36 or T318A complementary DNA (cDNA) infectious clones [20] by using appropriate primers (**S2 Table**; genomic sequence of isolate T36 at GenBank AY170468 and of isolate T318A at GenBank DQ151548). PCR products were then digested and cloned into a pBluescript vector. The Twin-Strep-tag (TST; IBA) was fused to the CTV proteins at the C-terminal. The new constructs were finally cloned into the pMOG binary vector. The empty vector, which only expresses the TST tag as a peptide, was used as a negative control. The resulting plasmids were named pMOG-p25-T36-tag, pMOG-p25-T318A-tag, pMOG-p20-T36-tag, pMOG-p20-T318A-tag, and pMOG-tag (**S3 Table**).

To perform the silencing suppression assays, the cDNAs of CTV p20 and p25 corresponding to the viral isolates T36 and T318A were inserted into a binary expression vector derived from pCLEAN-G181 [61] with and without a C-terminal TST tag. In these constructs, protein expression is driven by the *Cauliflower mosaic virus* 35S promoter and terminator, while the 5’ and 3’ untranslated regions are derived from the *Cowpea mosaic virus* RNA-2 [62]. The resulting plasmids were named p235CPMVZ-p25-T36, p235CPMVZ-p25-T318A, p235CPMVZ-p20-T36, p235CPMVZ-p20-T318A, p235CPMVZ-p25-T36-tag, p235CPMVZ-p25-T318A-tag, p235CPMVZ-p20-T36-tag, and p235CPMVZ-p20-T318A-tag (**S3 Table**). Plasmids used to express the green fluorescent protein (GFP) and the p19 RNA silencing suppressor of *Tomato bushy stunt virus* (TBSV) were previously described [63] and named pDGB-p19 and pMOG-GFP, respectively (**S3 Table**).

To perform the BiFC assays, the coding sequences of p20 and p25 (without the stop codon and the tag sequence) were amplified by PCR from the aforementioned pBluescript plasmids by using appropriate primers (attb label; **S2 Table**). The coding sequence of AGO1 from *N*. *benthamiana* was amplified by PCR from the pROK-AGO1 vector [63]. The full-length cDNA fragments (without stop codon) of AGO4 and TH9 from *N*. *benthamiana* were amplified by reverse transcription PCR from a total RNA preparation of wild-type plants. Subsequent PCRs allowed the addition of the external attb markers (**S2 Table**). Reverse transcriptions were performed using SuperScript retrotranscriptase (Thermo) and PCRs with Phusion high-fidelity DNA polymerase (Thermo). PCR products were cloned into the binary vectors pSPYNE or pPSYCE using Gateway technology (Invitrogen) for further BiFC assays. These empty vectors were used as negative controls. The resulting plasmids were named pSPYNE-p25-T36, pSPYNE-p25-T318A, pSPYNE-p20-T36, pSPYNE-p20-T318A, pSPYCE-AGO1, pSPYCE-AGO4, pSPYCE-TRX, and pSPYNE (**S3 Table**).

### Plant growth and agroinfiltration for proteomics

Plasmids pMOG-p25-T36-tag, pMOG-p25-T318A-tag, pMOG-p20-T36-tag, pMOG-p20-T318A-tag, and pMOG-tag were electroporated into *A*. *tumefaciens* strain C58C1. Transformed bacteria were grown overnight in LB medium at 28°C with shaking and appropriate antibiotics and were used to prepare the inocula for agroinfiltration [20]. *N*. *benthamiana* plants were grown (before and after agroinfiltration) in chambers maintained at a temperature of 25°C and a light/dark photoperiod of 16/8 h. The leaves of eight plants (three batches) were agroinfiltrated (3- to 4-week-old plants). The corresponding leaves were collected 3 days post-inoculation.

### Affinity purification

Protein complexes containing the tagged viral proteins (p20 and p25) were purified from 15 g of each batch of *N*. *benthamiana* agroinfiltrated leaves by affinity chromatography run in native conditions by using 1 mL Strep-Tactin superflow columns (IBA), as previously performed [23]. Briefly, tissues were homogenized with liquid nitrogen in a mortar with an extraction buffer and a protease inhibitor cocktail (cOmplete; Roche). Protein purification was conducted using the ÄKTAprime plus liquid chromatography system (GE Healthcare) at 4°C and a flow rate of 1 mL/min. Fractions of bound protein complexes, eluted with an extraction buffer containing D-desthiobiotin, were analyzed by western blot with an anti-Strep-tag antibody (StrepMAB-Classic, HRP conjugate; IBA). Selected enriched fractions were pooled and proteins were precipitated.

### Mass spectrometry

Protein preparations were separated by sodium dodecyl sulfate-based polyacrylamide gel electrophoresis (SDS-PAGE; 12.5% polyacrylamide, 0.05% SDS). The bands corresponding to each sample were excised, cut into pieces, in-gel digested with sequencing-grade trypsin (Promega), and the resulting peptides were eluted. Then, liquid chromatography and tandem mass spectrometry (LC-MS/MS) was performed, and the peptides were analyzed in a mass spectrometer nanoESI qQTOF (5600 TripleTOF; ABSciex), as previously performed [23]. Finally, the ProteinPilot v4.5 (ABSciex) and Mascot v2.2 (Matrix Science) search engines were used for protein identification.

### Characterization of silencing suppression

*A*. *tumefaciens* GV3101 was individually transformed with the plasmids pMOG-GFP (to express GFP), p235CPMVZ-p25-T36, p235CPMVZ-p25-T318A, p235CPMVZ-p20-T36, p235CPMVZ-p20-T318A, p235CPMVZ-p25-T36-tag, p235CPMVZ-p25-T318A-tag, p235CPMVZ-p20-T36-tag, p235CPMVZ-p20-T318A-tag (to express the different versions of CTV p20 and p25), pDGB-p19 (to express TBSV p19 and serve as a positive control), and an empty plasmid (to serve as a negative control). The resulting strains were induced for 2 h at 28°C in 10 mM MES-NaOH (pH 5.6), 10 mM MgCl_2_, and 150 μM acetosyringone at an optical density of 0.5 (600 nm). For local silencing analysis, *N*. *benthamiana* leaves were infiltrated with a mixture of *A*. *tumefaciens* cultures (1:1) to express GFP and a viral suppressor. In general, transient GFP expression declines after some days due to RNA silencing, and the presence of a viral suppressor restrains this decline. Plants were cultivated at 25°C in a growth chamber and the infiltrated tissues were harvested after 6 days for GFP analysis. For systemic silencing analysis, the upper leaves of the same plants were infiltrated with *A*. *tumefaciens* to express only GFP at an optical density of 0.5 three days after the initial infiltration. In this case, GFP was subject to both local and systemic silencing [64] and the infiltrated tissues were harvested three days later. GFP was extracted by homogenizing harvested plant tissue in ten volumes of 0.1 M sodium carbonate (pH 9.6). GFP fluorescence was measured (excitation at 485 nm) by using a VICTOR X5 plate fluorimeter (Perkin-Elmer). Additional photos of the leaves were taken with a confocal microscope (Leica).

### Ortholog identification

A general bioinformatic pipeline (implemented in Biopython) [65] was developed to map any protein from one host, typically obtained by proteomics, to the ortholog gene of another better-annotated host. In particular, we applied this tool to perform the mapping of green plant proteins (hits detected by Mascot, with GenInfo Identifier [GI]) to individual *A*. *thaliana* genes. To achieve this, we used The Arabidopsis Information Resource (TAIR) release 10. By using the exponentially modified protein abundance index (emPAI) for the estimation of absolute protein amount in our samples [26], our tool first selected those hits with a differential value of ΔemPAI ≥ 0.1 (tissues expressing the CTV proteins p20 and p25 from isolates T36 and T318A versus control tissues). An emPAI = 0 was assumed for elements not appearing in the control tissues. Note that a value of 0.1 approximately corresponds to the median of the distributions in ΔemPAI for the hits identified by this technique. In this way, we removed background proteins that systematically appear in proteomic analyses, as well as proteins with very low abundance. Upon assessing the emPAI distributions, we found a rough exponential distribution, without bimodality, which indicates that the higher the abundance, the lower the number of proteins in the sample. Subsequently, our tool performed a sequence alignment using the Basic Local Alignment Search Tool (BLAST) [66] to obtain the corresponding ortholog in *A*. *thaliana*. Using the GI identifier, the corresponding amino acid sequence was retrieved from the NCBI database in FASTA format using the Entrez module of Biopython. For each hit, the tool then performed a protein alignment to obtain the precursor gene in *A*. *thaliana* and provided genes and their functional descriptions as outputs. Finally, only the alignments with e-values of < 0.0001 were considered, and repeated elements were removed (**S1 Data**). Open source software deposited in https://github.com/silsgs/protein2gene.

### Functional analysis

With both lists of p20- and p25-targeted host proteins (both isolates), two functional analyses were performed by using agriGO [67]. These served to identify the biological processes over-represented for each viral protein. The statistical significance, of each list with respect to the complete plant genome (TAIR release 9), was evaluated by a Fisher’s exact test (2x2 contingency tables) with a correction for multiple testing using the Benjamini-Hochberg’s false discovery rate (FDR) procedure (adjusted *P* < 0.05), only considering GO terms with five or more mapping entries. Using these identified biological processes, a functional network was constructed by using REVIGO [68] in which functions sharing elements were connected. These networks allowed to identify modules. Then, the specific lists of protein targets (relative to p20 from T36, p20 from T318A, p25 from T36, and p25 from T318A) were used for a functional re-analysis. This information highlighted the effect of each CTV isolate over these networks (**S3 Data**).

### Network analysis

The *A*. *thaliana* interactome was used to contextualize the host proteins identified as targets for the viral proteins. This interactome was constructed by accounting for all known interactions with experimental evidence [39,40]. The entire *A*. *thaliana* interactome was represented by using Cytoscape [69] and an analysis of the global topological properties was performed (quantitative evaluation of the position of each protein). This included computation of the degree of connectivity (*k*), average shortest path (*l*), betweenness centrality (*b*), and clustering coefficient (*C*) for each node in the network. For each list of virus-targeted proteins (relative to p20 from T36, p20 from T318A, p25 from T36, and p25 from T318A), the distributions of those topological properties were represented as boxplots. Random lists of proteins were also generated to obtain control distributions of topological properties (we repeated this process 10 times). These control distributions, as well as the distributions corresponding to the total proteins of the interactome, were also represented as boxplots. The statistical significance of the results was assessed using Mann-Whitney’s *U*-tests (*P* < 0.05), by comparing the resulting distribution (of a given property) for each list of virus-targeted proteins against the distribution of the entire interactome or the one corresponding to a random list. This calculation was performed in MATLAB (MathWorks).

### Bimolecular fluorescence complementation

The pSPYNE and pSPYCE vector system (especially developed for plants) was used [47]. On the one hand, the pSPYNE vector was used to generate the fusions between the CTV proteins (p20 and p25) and YFP^N^ (N-terminal), knowing that N-terminal p25 fusions were not functional *in planta*. On the other hand, the pSPYCE vector was used to generate the fusions between the plant proteins (AGO1, AGO4, or TH9) and YFP^C^ (C-terminal). The resulting constructs (see **S3 Table**) were transiently expressed *in planta* with *A*. *tumefaciens*. The inocula consisted of a combination of three cultures (in a 1:1:0.5 ratio): one carrying the viral protein of interest (p20 from T36, p20 from T318A, p25 from T36, or p25 from T318A) in the pSPYNE vector, another with the host targeted protein in the pSPYCE vector, and another carrying the silencing suppressor p19 from TBSV to enhance transient expression [70]. Leaves were collected 2 days post-inoculation and were analyzed using a confocal microscope (Leica) to measure fluorescence using the protocol for GFP, which is also valid for YFP.

## Supporting information

S1 Fig A) Experimental results of GFP expression in *N*. *benthamiana* plants to analyze the effects of the different viral suppressors on local and systemic RNA silencing (error bars correspond to standard deviations; three replicates). *Statistical significance (Welch’s *t*-tests, *P* < 0.05). B) Representative images of plant leaves expressing GFP together with a silencing suppressor (in the local case, the silencing suppressor is co-expressed in the same tissue, while in the systemic case, it is expressed in another tissue). Scale bar, 5 mm.
(TIFF)

S2 Fig Detailed schematics of the algorithm developed to map and filter protein lists from one organism to another (ortholog identification). (TIFF)

S3 Fig Venn diagrams between the putative interactors of p20, p25 (CTV), and VPg (TEV) obtained by AP-MS. A) Comparison in case of a combined list of interactors from both CTV isolates. B) Comparison in case of isolate T36. C) Comparison in case of isolate T318A.
(TIFF)

S4 Fig A) Validation of additional protein-protein interactions by BiFC *in planta* using a split YFP system. B) Control BiFC assays.
(TIFF)

S1 Table Protein sequence alignments between the two p20s and the two p25s from CTV (from isolates T36 and T318A) considered in this work. (DOCX)

S2 Table Nucleotide sequences of the primer sets used in this work. (DOCX)

S3 Table List of plasmids used in this work. (XLSX)

S1 Data Raw lists of host plant proteins identified by AP-MS for all viral proteins (p20 from T36, p20 from T318A, p25 from T36, and p25 from T318A). The mapping between *N*. *benthamiana* and *A*. *thaliana* is also presented.
(XLSX)

S2 Data Final lists of plant proteins (*A*. *thaliana* orthologs) targeted by CTV p20 and p25 from AP-MS. The emPAI values are presented. In addition, classified lists of targeted proteins by specific or common interactors of p20 and p25, also indicating the isolate from which they were identified. In the case of common interactors, both isolates are indicated if at least they are associated with one contact.
(XLSX)

S3 Data Lists of GO terms associated with the identified interactors of p20 and p25, controlling for isolates. (XLSX)
