# Supplementary figures and images for: Molecular signatures of silencing suppression degeneracy from a complex RNA virus
Source: PLoS Comput Biol. 2021 Jun 28;17(6):e1009166. doi: 10.1371/journal.pcbi.1009166 (PMC8270454; doi:10.1371/journal.pcbi.1009166)

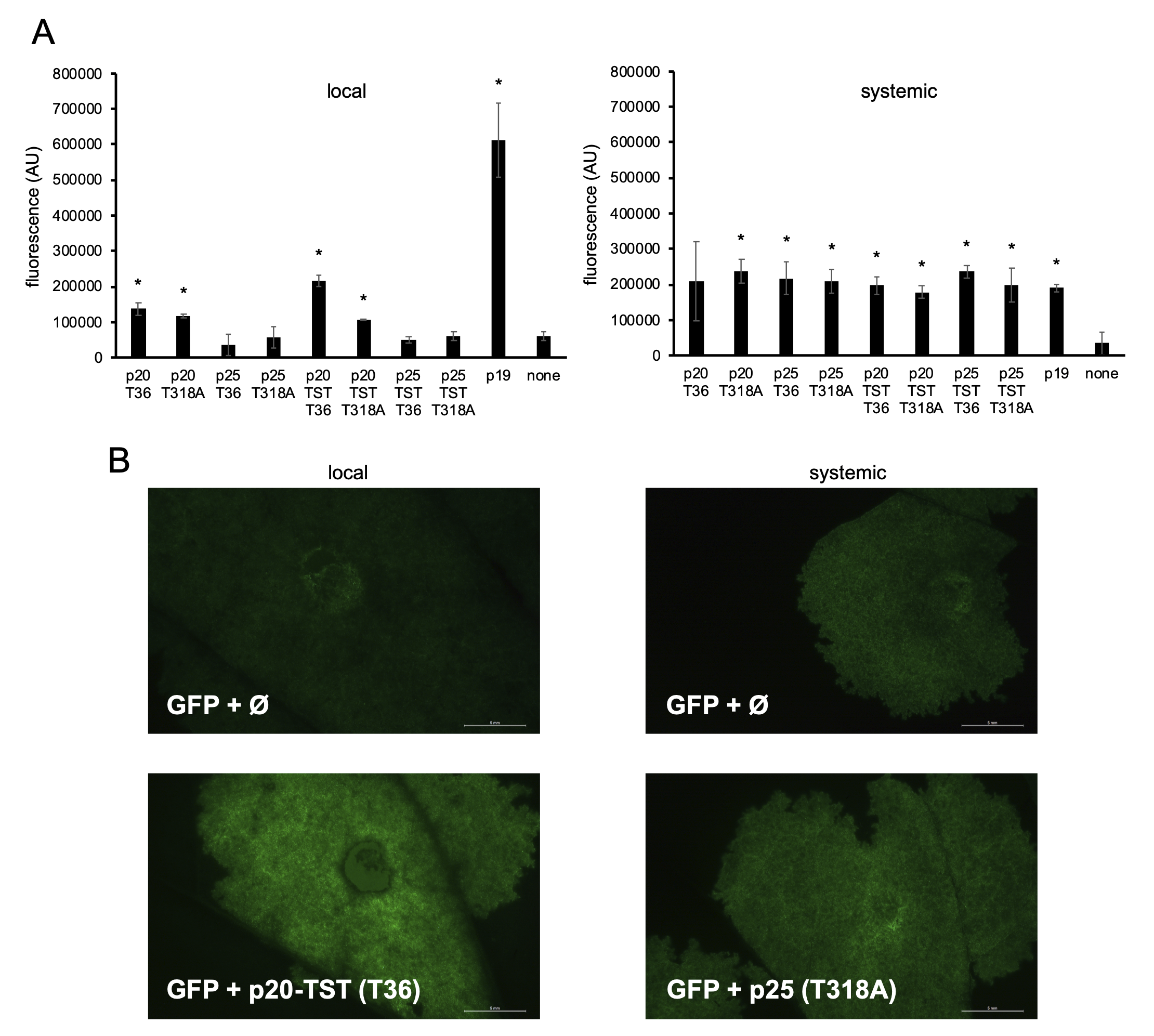

Supplement: S1 Fig — A) Experimental results of GFP expression in N. benthamiana plants to analyze the effects of the different viral suppressors on local and systemic RNA silencing (error bars correspond to standard deviations; three replicates). *Statistical significance (Welch’s t-tests, P < 0.05). B) Representative images of plant leaves expressing GFP together with a silencing suppressor (in the local case, the silencing suppressor is co-expressed in the same tissue, while in the systemic case, it is expressed in another tissue). Scale bar, 5 mm. (TIFF) [file pcbi.1009166.s001.tiff]

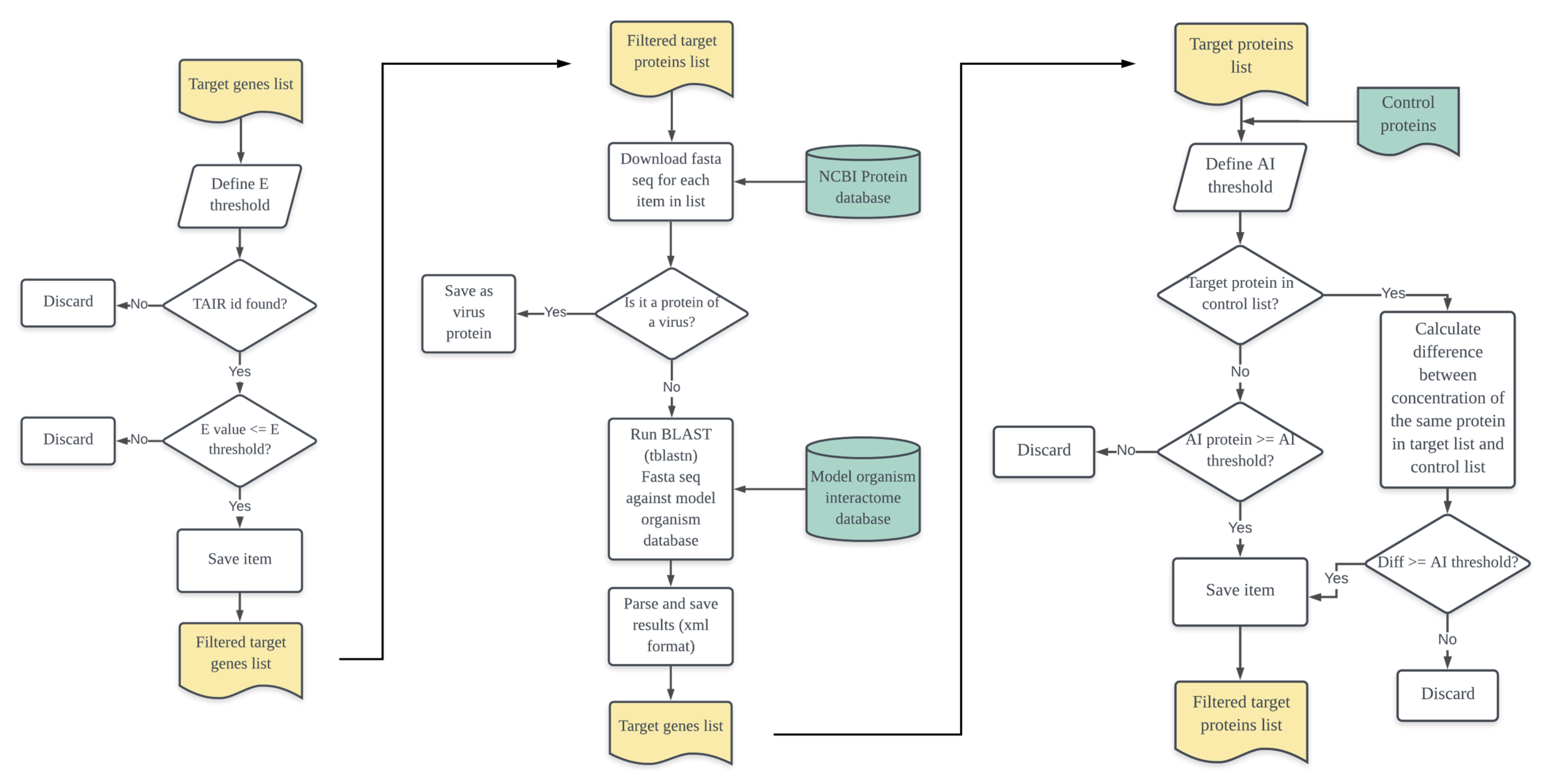

Supplement: S2 Fig — (TIFF) [file pcbi.1009166.s002.tiff]

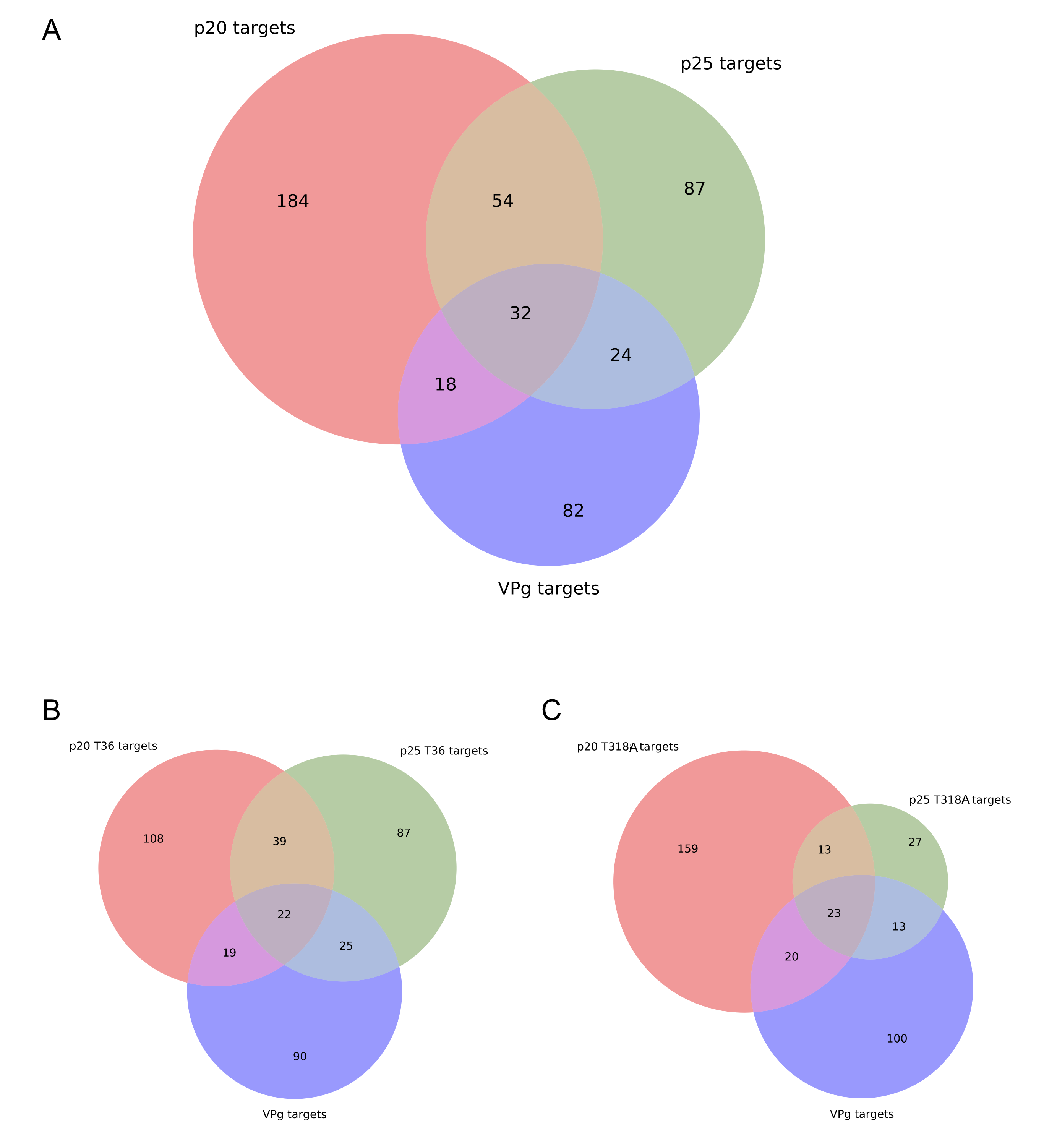

Supplement: S3 Fig — A) Comparison in case of a combined list of interactors from both CTV isolates. B) Comparison in case of isolate T36. C) Comparison in case of isolate T318A. (TIFF) [file pcbi.1009166.s003.tiff]

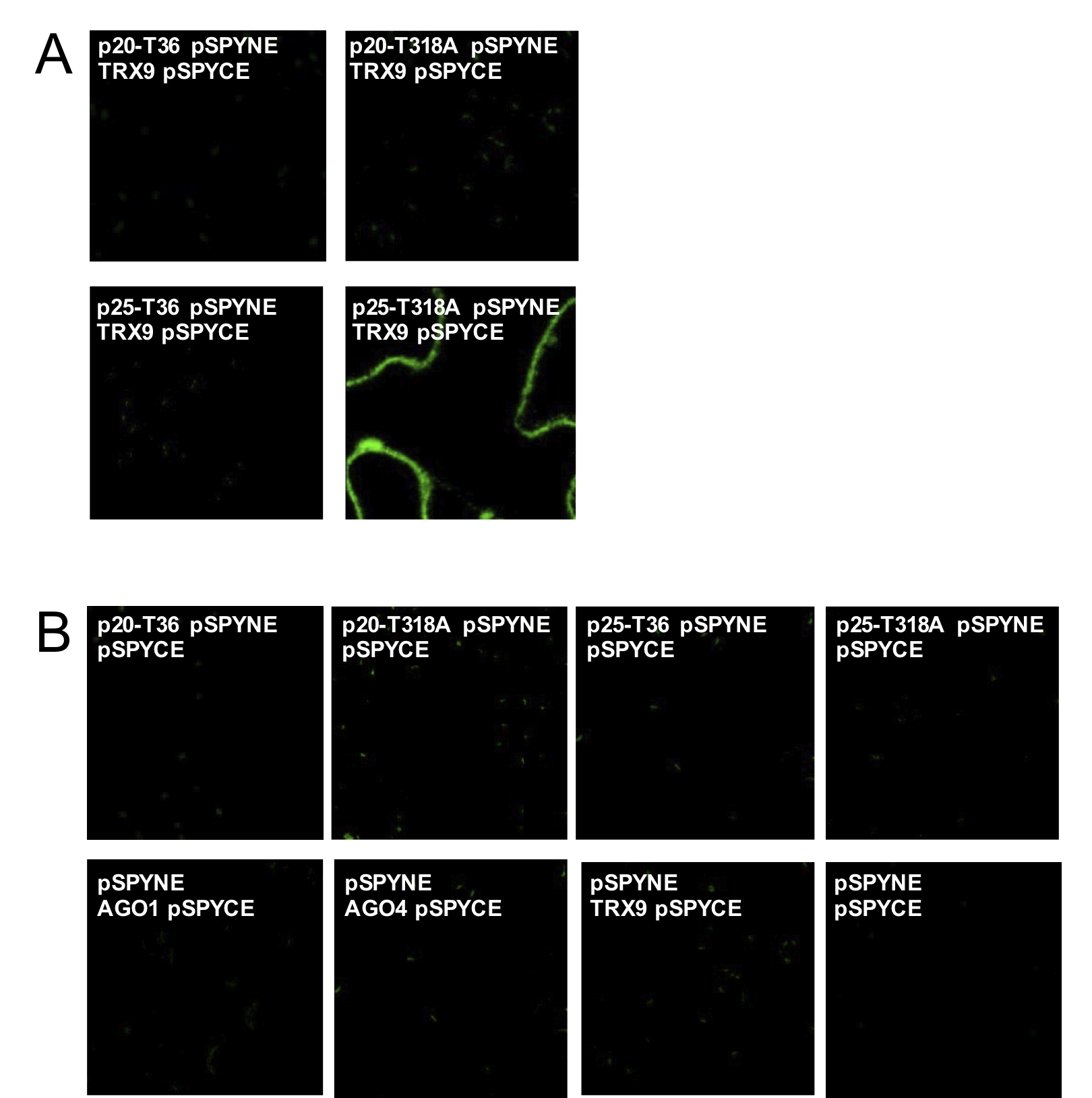

Supplement: S4 Fig — A) Validation of additional protein-protein interactions by BiFC in planta using a split YFP system. B) Control BiFC assays. (TIFF) [file pcbi.1009166.s004.tiff]
