# Supplementary material for: Molecular signatures of silencing suppression degeneracy from a complex RNA virus
Source: PLoS Comput Biol. 2021 Jun 28;17(6):e1009166. doi: 10.1371/journal.pcbi.1009166 (PMC8270454; doi:10.1371/journal.pcbi.1009166)
Supplement: S1 Table — (DOCX) [file pcbi.1009166.s005.docx]

| **Protein sequence alignments** |
| --- |
| p20_T36 1 MRAYFSVNDYISLLAKVSAVVERLCDPSVTLAEVMDEINDFNSFLALVHS 50  \|\|\|\|\|\|\|\|\|\|\|\|\|\|\|\|\|.\|\|\|\|\|\|\|\|\|\|\|\|\|.\|\|\|\|\|\|\|\|\|\|\|\|\|\|\|\|\|\|  p20_T318A 1 MRAYFSVNDYISLLAKVGAVVERLCDPSVTLTEVMDEINDFNSFLALVHS 50  p20_T36 51 MKSDMNGDHQDGHHEMGEHKSRLLCNIEAKLRVLLDIIRRRFTRDKLLCT 100  \|\|\|\|\|\|\|\|\|\|\|\|\|\|\|\|\|\|\|\|\|\|\|\|\|\|\|\|\|\|\|\|:\|\|\|\|\|\|\|\|\|\|\|\|\|\|\|\|\|  p20_T318A 51 MKSDMNGDHQDGHHEMGEHKSRLLCNIEAKLRILLDIIRRRFTRDKLLCT 100  p20_T36 101 SATDVMGFFVMRYMSSSHTSFESVMRTELRLVVKAVLSDLSRAHKLDFSE 150  \|\|\|\|\|\|\|\|\|\|\|\|\|\|\|\|\|\|\|\|\|\|\|\|\|\|\|\|\|:\|\|\|\|\|\|\|\|\|\|\|\|\|\|\|\|\|\|\|\|  p20_T318A 101 SATDVMGFFVMRYMSSSHTSFESVMRTELKLVVKAVLSDLSRAHKLDFSE 150  p20_T36 151 RAFAAYGILLQKGTVSTVCGQFDINLVSPSCV 182  \|\|\|\|\|\|\|\|\|\|\|\|\|\|\|:\|\|\|\|\|\|\|\|\|\|\|\|\|\|\|\|  p20_T318A 151 RAFAAYGILLQKGTVATVCGQFDINLVSPSCV 182 |
| p25_T36 1 MDDETKKLKNKNKETKEGDDVVAAESSFSSVNLHIDPTLITMNDVRQLST 50  \|\|\|\|\|\|\|\|\|\|\|\|\|\|\|\|\|\|\|\|\|\|\|\|\|\|\|\|.\|:\|\|\|\|\|\|\|\|\|.\|\|\|\|\|\|\|\|\|  p25_T318A 1 MDDETKKLKNKNKETKEGDDVVAAESSFGSLNLHIDPTLIAMNDVRQLST 50  p25_T36 51 QQNAALNRDLFLTLKGKHPNLPDKDKDFHIAMMLYRLAVKSSSLQSDDDA 100  \|\|\|\|\|\|\|\|\|\|\|\|\|\|\|\|\|:\|\|\|.\|\|\|\|\|\|\|\|\|\|\|\|\|\|\|\|\|\|\|\|\|\|\|\|\|\|\|.  p25_T318A 51 QQNAALNRDLFLTLKGKYPNLSDKDKDFHIAMMLYRLAVKSSSLQSDDDT 100  p25_T36 101 TGITYTREGVEVDLSDKLWTDVVFNSKGIGNRTNALRVWGRTNDALYLAF 150  \|\|\|\|\|\|\|\|\|\|\|\|\|\|\|\|\|\|\|\|\|\|\|\|\|\|\|\|\|\|\|\|\|\|\|\|\|\|\|\|\|:\|\|\|\|\|\|\|\|  p25_T318A 101 TGITYTREGVEVDLSDKLWTDVVFNSKGIGNRTNALRVWGRSNDALYLAF 150  p25_T36 151 CRQNRNLSYGGRPLDAGIPAGYHYLCADFLTGAGLTDLECAVYIQAKEQL 200  \|\|\|\|\|\|\|\|\|\|\|\|\|\|\|\|\|\|\|\|\|\|\|\|\|\|\|\|\|\|\|\|\|\|\|\|\|\|\|\|\|\|\|:\|\|\|\|\|\|  p25_T318A 151 CRQNRNLSYGGRPLDAGIPAGYHYLCADFLTGAGLTDLECAVYVQAKEQL 200  p25_T36 201 LKKRGADDVVVTNVRQLGKFNTR 223  \|\|\|\|\|\|\|:\|\|\|\|\|\|\|\|\|\|\|\|\|\|\|  p25_T318A 201 LKKRGADEVVVTNVRQLGKFNTR 223 |
