# Supplementary material for: Molecular signatures of silencing suppression degeneracy from a complex RNA virus
Source: PLoS Comput Biol. 2021 Jun 28;17(6):e1009166. doi: 10.1371/journal.pcbi.1009166 (PMC8270454; doi:10.1371/journal.pcbi.1009166)
Supplement: S2 Table — (DOCX) [file pcbi.1009166.s006.docx]

|  | | | |
| --- | --- | --- | --- |
|  |  |  |  |
| **Primer** | **Sequence 5’ - 3’** | **Sense** | **Position** |
| p20T-36D | **atg**CGAGCTTACTTTAGTGTTAATGA | F | 1 - 26^a^ |
| p20-T36R | TACACGCAAGATGGAGAGACTAAATTA | R | 521 - 548^a^ |
| p20-T318AD | **atg**CGAGCTTACTTTAGTGTTAAC | F | 1 - 24^b^ |
| p20-T318AR | TACACGCATAAGGAGAAACTAAATT | R | 522 - 548^b^ |
| p20-T36-attb1 | GGGGACAAGTTTGTACAAAAAAGCAGGCTAT**atg**CGAGCTTACTTTAGTGTTAATG | F | 1 - 26^a^ |
| p20T36-attb2 | GGGGACCACTTTGTACAAGAAAGCTGGGTACACGCAAGATGGAGAGACTAAA | R | 525 - 548^a^ |
| p20-T318A-attb1 | GGGGACAAGTTTGTACAAAAAAGCAGGCTAT**atg**CGAGCTTACTTTAGTGTTAACG | F | 1 - 24^b^ |
| p20-T318A-attb2 | GGGGACCACTTTGTACAAGAAAGCTGGGTACACGCATGAAGGAGAAACTAAA | R | 525 - 548^b^ |
| p25-T36-attb1 | GGGGACAAGTTTGTACAAAAAAGCAGGCTAT**atg**GACGACGAAACAAAGAAATTGAAGAA | F | 1 - 29^c^ |
| p25-T36-attb2 | GGGGACCACTTTGTACAAGAAAGCTGGGTAACGTGTGTTGAATTTCCCAAGCTGCCTG | R | 641 - 669^c^ |
| p25-T318A-attb1 | GGGGACAAGTTTGTACAAAAAAGCAGGCTAT**atg**GACGACGAGACAAAGAAATTGAAG | F | 1 - 27^c^ |
| p25-T318A-attb2 | GGGGACCACTTTGTACAAGAAAGCTGGGTAACGTGTGTTAAATTTCCCAAGCTGCCTG | R | 641 - 669^c^ |
| AGO1-attb1 | *GGGGACAAGTTTGTACAAAAAAGCAGGCTAT***atg**GTGCGGAAGAAGAGGAC | F | 1 - 21^d^ |
| AGO1-attb2 | *GGGGACCACTTTGTACAAGAAAGCTGGGTA*ATAAAACATAACCCTCTTAACATTC | R | 3131 - 3153^d^ |
| AGO4-RT | TTAACAAAAGAACATGGAACTGGAAACTTT | R | Flanking 3’ ORF^e^ |
| AGO4-D | **atg**GCTGAAGAAGATCCGAATGGAGCAGCAGAAG | F | 1 - 34^e^ |
| AGO4-R | ACAAAAGAACATGGAACAGGAAACTTTTTCCTCGAGCTTCGGAAGCTGGGGAACG | R | Flanking 3’ ORF^e^ |
| AGO4-attb1 | *GGGGACAAGTTTGTACAAAAAAGCAGGCTAT***atg**GCTGAAGAAGATAAGAATGGAGCA | F | 1 - 28^e^ |
| AGO4-attb2 | *GGGGACCACTTTGTACAAGAAAGCTGGGTA*ACAAAAGAACATGGAACTGGAAACTTTT | R | 2689 - 2717^e^ |
| TRX-RT | AAAAAGAGAGGAGGGGGGTTGAATATGG | R | Flanking 3’ ORF^e^ |
| TRX-D | TTAAATTGGAAGTTGATTTGTTTTC | F | Flanking 5’ ORF^e^ |
| TRX-R | TCAACATACAACTCTGAGAGCC | R | Flanking 3’ ORF^e^ |
| TRX-attb1 | *GGGGACAAGTTTGTACAAAAAAGCAGGCTAT***atg**GGGATTACTGATATGGTACA | F | 1pb - 23^f^ |
| TRX-attb2 | *GGGGACCACTTTGTACAAGAAAGCTGGGTA*CTGCGGTTGCGTCTCACATA | R | 437 - 457^f^ |
|  |  |  |  |
| F and R indicates forward and reverse CTV gRNA sequence sense, respectively | | | |
| Start codons are showed in bold lower case | |  |  |
| The *attb* sequences recombinant with the pSPYNE and pSPYCE vectors are underlined or underlined with italics, respectively | | | |
| ^a^ Nucleotide position relative to the genomic sequence of p20 from T36 isolate (GenBank AY170468) | | | |
| ^b^ Nucleotide position relative to the genomic sequence of p20 from T318A isolate (GenBank DQ151548) | | | |
| ^c^ Nucleotide position relative to the genomic sequence of p25 from isolates T36 and T318A isolate (GenBank AY170468 and DQ151548) | | | |
| ^d^ Nucleotide position relative to the genomic sequence of AGO1-1 from *N. benthamiana* (GenBank DQ321488.1) | | | |
| ^e^ Nucleotide position relative to the genomic sequence of AGO4-2 from *N. benthamiana* (GenBank DQ321491.1) | | | |
| ^f^ Nucleotide position relative to the genomic sequence of TRX-H9 (NCBI reference sequence XM_019375804.1) | | | |
